# Family intervention in schizophrenia: A case report

**DOI:** 10.1192/j.eurpsy.2024.1477

**Published:** 2024-08-27

**Authors:** E. Arroyo Sánchez, C. Díaz Mayoral, P. Setién Preciados

**Affiliations:** Hospital Universitario Príncipe de Asturias, Madrid, Spain

## Abstract

**Introduction:**

Schizophrenia is a chronic mental illness that has a lifetime prevalence worldwide of about 1% regardless of culture, social class and race. This implies that it affects a large number of families. Family therapy has been used for years as a promising approach to intervene with people suffering from such pathology. It has been shown that families with a high level of hostility, critical comments and over-involvement are related to a higher number of relapses in the family member diagnosed with schizophrenia.

**Objectives:**

The objectives are to examine whether systemic interventions could help to decrease the emotion expressed in these family members and thus decrease the number of relapses of patients as an alternative to pharmacological treatment.

**Methods:**

A case report and a literature review on the impact of family therapy in patients with a diagnosis of schizophrenia. The search strategy included keywords such as “family intervention”, “schizophrenia” and “systemic therapy”. Selection criteria included randomized controlled trials (RCTs) and meta-analyses published between 2010 and 2021. Studies focused on the impact of family intervention on symptom management, relapse prevention and general functioning were included.

**Results:**

The findings consistently demonstrated the effectiveness of family intervention in improving outcomes for people with schizophrenia. These interventions generally involved psychoeducation, communication skills training, problem-solving techniques, and emotional support for family members. Results showed significant reductions in symptom severity, decreased relapse rates, improved adherence to pharmacological treatment, and better overall functioning among people who received family intervention compared with those who received only standard care. In addition, family intervention was associated with reduced caregiver burden, improved family communication, as well as increased knowledge about schizophrenia and its management.

**Conclusions:**

Family intervention has become a valuable adjunctive treatment for people with schizophrenia. The findings of this review highlight its positive impact on symptom management, relapse prevention, and overall functioning. Family intervention offers a holistic approach that recognizes the importance of involving and supporting the family system in the treatment process. This intervention provides families with the tools and resources necessary to effectively cope with the challenges associated with schizophrenia and promotes a supportive and nurturing environment for the individual. Future research should focus on long-term outcomes and implementation of the family intervention in routine clinical practice.

**Disclosure of Interest:**

None Declared

